# The effect of gamified breastfeeding education on breastfeeding self-efficacy and ınfant feeding attitudes of pregnant women: a randomized controlled study

**DOI:** 10.1590/1806-9282.20251003

**Published:** 2026-05-08

**Authors:** Ozlem Ulku Bulut, Zehra Golbasi

**Affiliations:** 1 Lokman Hekim University, Faculty of Health Sciences – Ankara, Turkey.

**Keywords:** Gamification, Breastfeeding, Self efficacy, Health education, Infant nutrition, Nursing.

## Abstract

**OBJECTIVE:**

The aim of this study was to investigate the effect of gamified breastfeeding education on breastfeeding self-efficacy and infant feeding attitudes of pregnant women.

**METHODS:**

A total of 60 primiparous women in the third trimester of pregnancy were randomly assigned to experimental (n=30) and control (n=30) groups. Data were collected using the Sociodemographic and Obstetric Characteristics Form, Breastfeeding Self-Efficacy Scale-Antenatal Form, and the Iowa Infant Feeding Attitude Scale. The experimental group received a gamified breastfeeding education program designed by the researchers and developed based on Werbach and Hunter’s D6 Gamification Framework. The program was delivered via a secure Web 2.0 platform and included the interactive digital game “*Discovering Breastfeeding*” and an educational escape room-style activity enriched with visual and auditory elements. The intervention aimed to enhance participants’ motivation, engagement, and learning through game mechanics such as goals, feedback, challenges, and rewards. The control group received routine hospital antenatal education. Pretest and posttest data were analyzed using appropriate statistical methods.

**RESULTS:**

No significant difference was found between the groups in baseline measurements. However, post-test scores revealed that women in the experimental group showed a significant improvement in both breastfeeding self-efficacy and infant feeding attitudes compared to the control group (p<0.05). Effect sizes indicated a strong impact of the intervention.

**CONCLUSION:**

Gamified breastfeeding education based on digital and interactive learning principles effectively enhanced pregnant women’s self-efficacy and attitudes toward infant feeding. Incorporating gamification into maternal health education may increase motivation, promote positive behavioral change, and contribute to better breastfeeding outcomes for mothers and infants.

The trial was registered in the clinicaltrials.gov notification system (Clinical Trials Number: NCT06344806).

## INTRODUCTION

Breastfeeding has long been the cornerstone of infant nutrition, yet global rates remain suboptimal. The World Health Organization (WHO) recommends exclusive breastfeeding for the first 6 months and continued breastfeeding up to 2 years, but only 44% of infants worldwide meet the 6-month target; in Turkey, the rate is 41%^1^. To improve these rates, WHO and United Nations Children’s Fund (UNICEF) aim to exceed 70% exclusive breastfeeding globally by 2030^2,3^.

A key determinant of breastfeeding continuity is breastfeeding self-efficacy, defined as a mother’s confidence in her ability to breastfeed^4,5^. Higher self-efficacy is linked to longer duration and greater breastfeeding success^6,7^. It influences emotional responses and persistence in overcoming difficulties. In addition, attitudes toward infant feeding play a crucial role in shaping mothers’ feeding preferences and behaviors^8,9^. Positive attitudes have been shown to predict exclusive breastfeeding and longer continuation^10,11,12^. Therefore, interventions that strengthen both self-efficacy and feeding attitudes are essential for promoting breastfeeding success.

Recent educational research highlights the importance of digital and interactive methods in maternal health training. Gamification, defined as the use of game elements in non-game contexts, has emerged as a novel pedagogical approach that enhances motivation, engagement, and behavioral change^13^. In healthcare education, gamified and technology-supported learning environments have been found to increase knowledge retention and improve self-directed learning^14,[Bibr B15],16^. In the context of breastfeeding, gamified interventions may bridge existing knowledge gaps, create a sense of enjoyment in learning, and support decision-making through feedback, challenges, and reward mechanisms.

This randomized controlled study aimed to evaluate the effect of a gamified breastfeeding education program-implemented through a Web 2.0-based interactive platform—on the breastfeeding self-efficacy and infant feeding attitudes of pregnant women. This research contributes to the growing evidence base supporting innovative, technology-enhanced educational ­models in maternal and newborn health.

## METHODS

### Data collection

**Figure 1:**
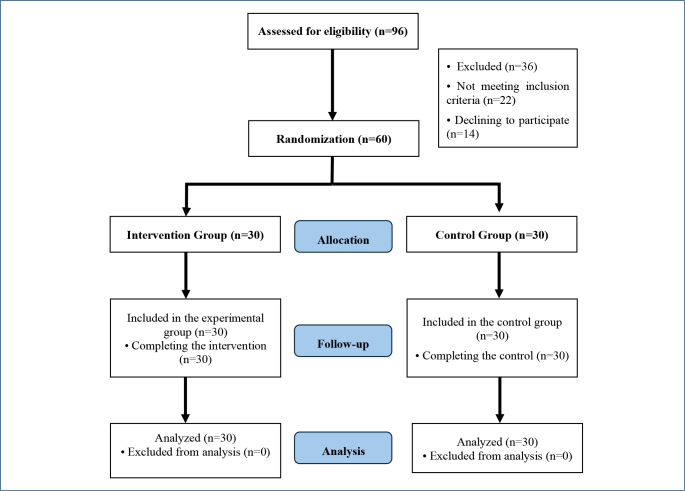
Consort flow diagram.

This study was a randomized controlled experimental study. Sixty women randomly selected from the population were assigned to either the experimental group (30 women) or the control group (30 women) through a block randomization process conducted by a statistical expert independent of the researcher. Inclusion criteria included primiparous mothers between 35 and 37 weeks of gestation who had access to a smartphone, tablet, or computer with an internet connection. Exclusion criteria included women with pregnancy risk factors, planned cesarean delivery, and contraindications to breastfeeding. Recommendations from the CONSORT group (Consolidated Standards of Reporting Trials) were followed in this study (Figure 1). This study is registered in The ClinicalTrials.gov Protocol Registration and Results System (PRS) ID: NCT06344806.

Sociodemographic and Obstetric Characteristics Assessment Form, Breastfeeding Self-Efficacy Scale Antenatal Form (pretest–post-test) and IOWA Infant Feeding Attitudes Scale (pretest–post-test) were used in the study. The reliability coefficient of the IOWA scale was calculated as 0.859, and the Breastfeeding Self-Efficacy Scale Antenatal Form was calculated as 0.889 in this study.

### Gamified breastfeeding counseling program

The training program incorporated the “Discovering Breastfeeding” game developed based on Werbach and Hunter’s D6 Gamification Framework. The gamified education included a single 20-min online session conducted between the 35th and 37th weeks of pregnancy. The session was implemented through a secure Web 2.0 platform (Genially) and consisted of an interactive digital game and an escape room–style educational activity that combined visual and auditory elements. Within the D6 framework, each gamification element was purposefully operationalized: challenges required solving breastfeeding-related scenarios, feedback was provided instantly through digital prompts and verbal reinforcement, and rewards were represented by virtual achievement badges displayed at the end of each section. These elements aligned with the theoretical dimensions of motivation, progression, and reinforcement within the D6 model.

Escape Room games are immersive and enjoyable activities that require participants to solve educational puzzles and achieve specific objectives within a defined scenario. In this study, these puzzles were designed around key breastfeeding topics such as latch technique, milk production, and common breastfeeding challenges. The aim was to enhance motivation, engagement, and knowledge retention through game elements such as goals, feedback, challenges, and rewards. Specifically, challenges involved solving breastfeeding-related scenarios, feedback was provided instantly through digital prompts, and rewards were represented by virtual achievement badges. The intervention was delivered by the principal researcher, who had expertise in maternal health education and gamification design.

The control group received standard prenatal care and education provided routinely by the hospital. The post-test data were collected immediately after the completion of the single gamified education session in the experimental group and after routine education in the control group.

### Interventions applied to the control group

In this study, participants in the control group received prenatal care provided by the hospital. This care included regular prenatal evaluations throughout pregnancy. The control group received no additional therapies, and their care followed the regular protocols available to all pregnant women in the hospital, minus the gamified breastfeeding guidance provided to the experimental group. The outcomes of the control group were juxtaposed with those of the experimental group to assess the efficacy of the gamified breastfeeding counseling intervention.

### Intervention

Pretest (IOWA and Breastfeeding Self-Efficacy Scale-Antenatal Form) was administered to primiparous pregnant women in the last trimester who agreed to participate in the study. Randomization was then performed. Verbal and written informed consent was obtained from 60 women assigned to the experimental and control groups. A gamified breastfeeding education program was applied to 30 women in the experimental group. The 30 women in the control group received routine care at the hospital. After the training program, the post-test (IOWA and Breastfeeding Self-Efficacy Scale-Antenatal Form) was administered to the experimental group.

### Ethical considerations

The study was granted ethical approval by the Scientific Research Ethics Committee (Decision no: 2022/1-1) for its implementation. All participants who met the inclusion criteria provided informed written consent. Both groups were provided with the standard care that is given to all women in the hospital. Following the completion of the trial, a gamified breastfeeding counseling program was implemented for the women in the control group in order to uphold the idea of equality.

### Statistical analysis

Data were analyzed using Statistical Package for the Social Sciences (SPSS) 22.0. Descriptive statistics included number, percentage, mean, and standard deviation. Normality was assessed via skewness and kurtosis, with values between ±1.5 and ±2.0 considered acceptable^14,15^. All variables were normally distributed. Categorical variables were analyzed using chi-square or Fisher’s exact test. The independent samples t-test compared continuous variables between groups, while the paired samples t-test assessed intra-group changes. Effect sizes (Cohen’s d) were calculated to interpret the magnitude of the observed effects. Statistical significance was set at p<0.05.

### Limitations of the study

- Blinding was not feasible, as participants were aware of the intervention.
- Conducted in a private hospital with participants from a high socioeconomic background, limiting generalizability.
- The study was limited to the antenatal period; no postnatal follow-up was performed.
- Excluded women without access to digital devices or the internet.

### Strengths of the study

- Employed a randomized controlled design with impartial statistical analysis.
- Used validated and reliable measurement tools.
- Introduced an innovative “Gamified Breastfeeding Counseling Program” within Women’s Health Nursing in Turkey.

## RESULTS

No statistically significant differences were found between the experimental and control groups in terms of education, employment, income level, family type, planned pregnancy, or intended breastfeeding duration (p>0.05). Most participants were university graduates, unemployed, from nuclear families, and reported income equal to expenses. The majority intended to breastfeed for up to 24 months. Age and gestational week distributions were also similar between the groups, confirming baseline comparability (Table 1).

**Table 1:** Scores of the intervention and control groups from the Iowa Infant Feeding Attitude Scale.

Groups	Intervention (n=30)		Control (n=30)		t^a^	P
	Mean	SD	Mean	SD		
IOWA total pretest	65.867	6.816	64.333	6.031	0.923	0.360
IOWA total post-test	70.467	4.392	64.033	6.467	4.507	**0.000**
t_b_	-4.873		0.717			
P	**0.000**		0.479			

^a^Independent groups t-test

^b^Dependent groups t-test. SD: standard deviation; IOWA: Iowa Infant Feeding Attitude Scale. Bold values in the tables indicate statistically significant results (p<0.05).

There was no significant difference between groups in infant feeding attitude scores at pretest (p>0.05). However, post-test scores were significantly higher in the experimental group ( $\bar{\text{x}}$ =70.47) than in the control group ( $\bar{\text{x}}$ =64.03) [t(58)=4.507; p>0.001]. A significant increase was observed in the experimental group from pretest ( $\bar{\text{x}}$ =65.87 to post-test (t=-4.873; p>0.001; d=0.890), while the change in the control group was not statistically significant (p>0.05).

Post-test scores on the breastfeeding self-efficacy scale were significantly higher in the experimental group ( $\bar{\text{x}}$ =56.63) compared to the control group ( $\bar{\text{x}}$ =49.37) [t(58)=2.509; p=0.015]. No significant difference was observed between groups at pretest (p>0.05). Within the experimental group, a significant increase was noted from pretest to posttest ( $\bar{\text{x}}$ =49.77 → $\bar{\text{x}}$ =56.63; t=-4.313; p>0.001; d=0.787). Although the control group also showed a significant increase ( $\bar{\text{x}}$ =47.47 → $\bar{\text{x}}$ =49.37; t=-2.148; p=0.040; d=0.392), the effect size was smaller. Breastfeeding self-efficacy scores of the intervention and control groups are presented in Table 2.

**Table 2:** Breastfeeding self-efficacy scale antenatal form scores of experimental and control groups

Groups	Intervention (n=30)		Control (n=30)		t^a^	p
	Mean	SD	Mean	SD		
Breastfeeding self-efficacy pre-test	49.767	11.288	47.467	10.922	0.802	0.426
Breastfeeding self-efficacy post-test	56.633	11.625	49.367	10.797	2.509	**0.015**
t^b^	-4.313		-2.148			
P	**0.000**		**0.040**			

^a^Independent groups t-test

^b^Dependent groups t-test. SD: standard deviation. Bold values in the tables indicate statistically significant results (p<0.05).

Post-test measurements were obtained immediately after the completion of the gamified breastfeeding education program in the experimental group and after routine antenatal education in the control group.

## DISCUSSION

The findings of this study indicate that gamified breastfeeding education significantly improved both breastfeeding self-efficacy and infant feeding attitudes among pregnant women. The lack of significant differences in pretest scores confirmed baseline similarity, while posttest scores demonstrated a substantial increase in both outcomes for the experimental group, supported by large effect sizes (d=0.890 for infant feeding attitude; d=0.787 for self-efficacy).

These results align with previous studies emphasizing the effectiveness of interactive and engaging educational strategies in maternal health. For instance, Grassley et al. and Öztürk et al. demonstrated that technology-supported, game-based learning can positively impact breastfeeding outcomes^11,12^. Similarly, Shafaei et al. showed that prenatal counseling increased self-efficacy and reduced breastfeeding problems, while Simsek-çetinkaya et al. reported improved self-efficacy and attitudes via a nurse-led digital education program^16,17^. Recent systematic reviews have further confirmed that digital and gamified interventions in maternal health improve exclusive breastfeeding duration and maternal confidence^18,19^. These results reinforce the integration of gamified strategies into maternal health education, particularly during the prenatal period.

Tseng et al. also found that a structured breastfeeding intervention grounded in self-efficacy theory significantly improved breastfeeding outcomes across multiple time points, including postpartum^20^. Although our study focused only on the antenatal period, similar positive outcomes were observed immediately after the intervention. Comparable findings were also reported in a recent digital intervention meta-analysis, where interactive and feedback-based education significantly enhanced breastfeeding continuation up to 6 months postpartum^21^. In our study, the control group showed a minor increase in self-efficacy scores (d=0.392), after the routine antenatal education, yet no improvement in feeding attitudes, indicating that traditional methods may provide limited benefits compared to the gamified program.

Moreover, previous research has shown that factors such as family structure, education, income, and initial breastfeeding intention influence breastfeeding outcomes^8,9^. Since these variables were evenly distributed between the groups in our study, the improvements observed can be attributed to the gamified intervention rather than external factors. This aligns with recent evidence suggesting that technology-based breastfeeding education reduces sociodemographic disparities by providing equitable access to learning opportunities and support^22^.

In conclusion, gamified education appears to be an effective approach in antenatal training. By enhancing motivation, engagement, and learning outcomes, gamification supports behavior change and empowers women in their breastfeeding journey. These results reinforce the integration of gamified strategies into maternal health education, consistent with existing literature^6,11,23^.

## CONCLUSION

This study demonstrated that a gamified breastfeeding education program effectively improved pregnant women’s breastfeeding self-efficacy and their attitudes toward infant feeding. The experimental group that received the gamified intervention showed significantly higher posttest scores compared to the control group receiving routine antenatal education. These findings are consistent with previous research emphasizing the benefits of innovative, interactive, and engaging educational approaches in enhancing health-related self-efficacy and attitudes.

Gamified strategies appear to be effective tools in maternal health education, particularly for supporting breastfeeding promotion during the antenatal period. By addressing knowledge gaps and boosting confidence, gamified education can foster positive behavioral change and contribute to improved breastfeeding outcomes for both mothers and infants. Future studies should investigate the long-term effects of gamified interventions and their applicability across different populations and cultural contexts.

## Data Availability

The datasets generated and/or analyzed during the current study are available from the corresponding author upon reasonable request.

## References

[1] Hacettepe University Institute of Population Studies (2019). 2018 Turkey demographic and health survey.

[2] World Health Organization (2019). Essential nutrition actions: mainstreaming nutrition through the life-course.

[3] World Health Organization, United Nations Children’s Fund (2022). Global breastfeeding scorecard, 2022: tracking progress for breastfeeding policies and programmes.

[4] Galipeau R, Baillot A, Trottier A, Lemire L (2018). Effectiveness of interventions on breastfeeding self-efficacy and perceived insufficient milk supply: a systematic review and meta-analysis. Matern Child Nutr.

[5] Iliadou M, Lykeridou K, Prezerakos P, Swift EM, Tziaferi SG (2018). Measuring the effectiveness of a midwife-led education programme in terms of breastfeeding knowledge and self-efficacy, attitudes towards breastfeeding, and perceived barriers of breastfeeding among pregnant women. Mater Sociomed.

[6] Aktürk NBK, Kolcu M (2023). The effect of postnatal breastfeeding education given to women on breastfeeding self-efficacy and breastfeeding success. Rev Assoc Med Bras (1992).

[7] Cardoso A, Silva A, Marin H (2017). Pregnant women’s knowledge gaps about breastfeeding in northern Portugal. Open J Obstet Gynecol.

[8] Renuka M, Shabadi N, Kulkarni P, Kumar D, Anup G, Murthy M (2020). Effectiveness of educational intervention on breastfeeding among primi pregnant women-a longitudinal study. Clin Epidemiol Glob Health.

[9] Rahmadani A, Rahmawati A (2022). Meta-analysis: effect of breastfeeding education program on the breastfeeding self-efficacy and exclusive breastfeeding. J Health Promot Behav.

[10] Karaahmet AY, Bilgiç FŞ (2022). Breastfeeding success in the first 6 months of online breastfeeding counseling after cesarean delivery and its effect on anthropometric measurements of the baby: a randomized controlled study. Rev Assoc Med Bras (1992).

[11] Öztürk R, Ergün S, Özyazıcıoğlu N (2022). Effect of antenatal educational intervention on maternal breastfeeding self-efficacy and breastfeeding success: a quasi-experimental study. Rev Esc Enferm USP.

[12] Grassley JS, Connor KC, Bond L (2017). Game-based online antenatal breastfeeding education: a pilot. Appl Nurs Res.

[13] Werbach K, Hunter D (2015). The Gamification toolkit: dynamics, mechanics, and components for the win.

[14] Tabachnick BG, Fidell LS (2013). Using multivariate statistics.

[B15] George D, Mallery M (2010). SPSS for windows step by step: a simple guide and reference, 17.0 update.

[16] Shafaei FS, Mirghafourvand M, Havizari S (2020). The effect of prenatal counseling on breastfeeding self-efficacy and frequency of breastfeeding problems in mothers with previous unsuccessful breastfeeding: a randomized controlled clinical trial. BMC Womens Health.

[17] Şimsek-Çetinkaya Ş, Gümüş Çaliş G, Kibris Ş (2024). Effect of breastfeeding education program and nurse-led breastfeeding online counseling system (BMUM) for mothers: a randomized controlled study. J Hum Lact.

[18] Thepha T, Carr G, Marais D, Kuasri J, Klangphaow K, Tangpukdee J (2024). The effectiveness of digital health versus standard care on exclusive breastfeeding duration among postpartum mothers in LMIC: systematic review and meta-analysis. Digit Health.

[19] Patnode CD, Senger CA, Coppola EL, Iacocca MO (2025). Interventions to support breastfeeding: updated evidence report and systematic review for the US Preventive Services Task Force. JAMA.

[20] Tseng JF, Chen SR, Au HK, Chipojola R, Lee GT, Lee PH (2020). Effectiveness of an integrated breastfeeding education program to improve self-efficacy and exclusive breastfeeding rate: a single-blind, randomised controlled study. Int J Nurs Stud.

[21] Iamchareon T, Maneesriwongul W (2025). The effectiveness of real-time telelactation intervention on breastfeeding outcomes among employed mothers: a systematic review and meta-analysis. BMC Pregnancy Childbirth.

[22] Laws RA, Cheng H, Rossiter C, Kuswara K, Markides BR, Size D (2023). Perinatal support for breastfeeding using mHealth: a mixed methods feasibility study of the My Baby Now app. Matern Child Nutr.

[23] Hartati S, Hakim N (2021). A new exclusive breastfeeding booklet to improve self-efficacy. KnE Life Sci.

